# Impact of Empagliflozin on the Outcomes of β-Thalassemia Major in Patients With Type 2 Diabetes Mellitus: The THALEMPA Observational Study

**DOI:** 10.7759/cureus.69837

**Published:** 2024-09-21

**Authors:** Anas Ibraheem, Waseem F Al Tameemi

**Affiliations:** 1 Department of Hematology, King's College Hospital, London, GBR; 2 Department of Internal Medicine, Section of Hematology, Al-Nahrain University College of Medicine, Baghdad, IRQ; 3 Department of Internal Medicine, Section of Hematology, Al-Immamain Al-Kadhumein Medical City, Baghdad, IRQ

**Keywords:** beta-thalassemia major, diabetes mellitus type 2, empagliflozin, hyperuricemia, secondary iron overload, sodium-glucose cotransporter-2 (sglt2) inhibitors, transfusion-dependent thalassemia (tdt)

## Abstract

Objective: Beta-thalassemia major (β-TM) is a genetic disorder characterized by ineffective erythropoiesis and chronic hemolytic anemia, necessitating lifelong blood transfusions and leading to severe complications. This study, termed THALEMPA by the authors, investigated the effect of empagliflozin (EMPA) on β-TM outcomes in patients with type 2 diabetes mellitus (T2DM), focusing on disease severity and associated complications of iron overload and hyperuricemia.

Methodology: This study conducted a single-center prospective observational investigation involving adults diagnosed with β-TM and T2DM. A total of 20 carefully selected patients were stratified into two groups based on their medical condition: the EMPA group, receiving 10 mg of empagliflozin, and a control group, receiving standard care. This focused cohort size was chosen to ensure a detailed, in-depth analysis of the treatment effects within this specific patient population. Over three months, both groups were closely monitored for β-TM outcomes. The study assessed β-TM severity parameters such as hemoglobin levels, blood transfusion frequency, aspartate aminotransferase (AST), alanine aminotransferase (ALT), left ventricular ejection fraction percentage, and spleen size. Additionally, β-TM complications were evaluated through serum ferritin and uric acid levels.

Results: Our analysis revealed that EMPA increased hemoglobin levels by up to 0.56 g/dL compared to baseline (*P *< 0.05). Liver enzyme levels significantly improved with EMPA by the third month. AST and ALT decreased by 36.22% and 33.36%, respectively, from baseline levels (*P *< 0.05), highlighting EMPA's potential benefits for β-TM severity. Serum ferritin and uric acid levels decreased by 27.93% and 21.29%, respectively, over three months on EMPA (*P *< 0.05). However, other parameters did not show significant changes post-EMPA.

Conclusions: This study demonstrates the significant impact of EMPA treatment over three months on β-TM patients with T2DM, evidenced by notable improvements in hemoglobin levels and reductions in liver enzymes, as well as in complications related to iron overload and hyperuricemia. Future research should confirm these benefits over longer durations and assess broader patient outcomes such as quality of life.

## Introduction

β-Thalassemia is an autosomal recessive disorder characterized by a reduction or absence in synthesizing one or more β-globin chains, leading to ineffective erythropoiesis and chronic hemolytic anemia [1]. It exhibits epidemiological regional variation across the globe, with the highest occurrence observed in the Mediterranean, the Middle East, Southeast Asia, and Central Asia. Approximately 68,000 infants are born with the disease each year, and roughly 80-90 million people are carriers, constituting approximately 1.5% of the world's population. [2,3]. Beta-thalassemia major (β-TM), or *transfusion-dependent thalassemia*, is the most severe form of the disease. It is characterized by the presence of two defective or absent β-globin genes. It requires regular and lifelong transfusions of red blood cells, leading to a wide range of complications that are either related to frequent blood transfusions or the disease per se [4]. These complications include iron overload, damage to vital organs, osteoporosis, growth retardation, heart and endocrine system dysfunctions, and a higher risk of premature mortality [5-8].

β-TM is typically diagnosed in early childhood and confirmed through laboratory tests. While the standard care for most β-TM patients worldwide involves regular blood transfusions and chelating agents, other methods such as bone marrow and stem cell transplantation, gene therapy, and induction of fetal hemoglobin production are available [5,9-11]. Despite the available treatment choices, there is a demand for a cost-effective solution capable of delivering favorable results by minimizing the necessity for frequent blood transfusions and mitigating ensuing complications. Herein, we explore the potential of empagliflozin (EMPA), a sodium-glucose cotransporter 2 inhibitor (SGLT2), to impact the disease outcome significantly. SGLT2 inhibitors, including canagliflozin, dapagliflozin, EMPA, and ertugliflozin, have been FDA-approved for managing adult patients with type 2 diabetes mellitus (T2DM). They act at the forefront of renal glucose reabsorption, leading to glucose homeostasis independently of insulin action [12,13]. Beyond glycemic control, SGLT2 inhibitors have shown impressive cardio-renal benefits and a modest reduction in body weight and blood pressure in recent years [14-17]. Imagine a scenario where SGLT2 inhibitors can treat β-TM with remarkable results.

Although this topic in medicine is still raw, the existing body of literature furnishes valuable insights and comprehensive information that these inhibitors can be a promising breakthrough treatment for β-TM patients, increasing hemoglobin levels and decreasing the blood transfusion rate, iron overload, and subsequent complications [18-22]. Several studies, including post hoc mediation analyses from the EMPA‐REG OUTCOME trial and CANVAS Program, have shown that SGLT2 inhibitors can cause an initial increase in erythropoietin (EPO) levels, leading to a rise in hematocrit, hemoglobin, and soluble transferrin receptor levels, as well as counteracting serum ferritin, hepcidin, and iron overload [14-16,23,24]. In addition, a recent study by Schwarz et al. reported that the administration of SGLT2 inhibitors was linked to a notable increase in hematocrit levels, polycythemia, and a reduced prevalence of anemia [25]. Thalassemia patients have a higher cell turnover rate due to chronic hemolysis and ineffective erythropoiesis. This makes them more liable to develop hyperuricemia, a common complication of β-TM [26]. Several studies have shown that SGLT2 inhibitors possess properties that can reduce hyperuricemia through various mechanisms [27-29].

The effects of EMPA were prospectively observed on the outcomes of β-TM patients with T2DM, particularly the disease severity and the associated complications of iron overload and hyperuricemia. This study was termed THALEMPA by the authors. The findings of this study could contribute to advancing tailored therapies to reduce complications and enhance outcomes for β-TM patients with T2DM.

## Materials and methods

Study oversight

The study complied with the Declaration of Helsinki and the International Conference on Harmonization Good Clinical Practice (ICH GCP) Guidelines and received approval from the Research Ethical Committee of Al-Nahrain University College of Medicine (IRB No. 20240237). All patients provided informed consent, ensuring confidentiality and privacy. A specialized medical team monitored patients regularly for severe adverse events, including death, ketoacidosis, hypotension, genitourinary infections, hospitalization, acute kidney injury, thrombosis, significant disability, or other medically significant events. If adverse effects arose, the drug would be withdrawn under a diabetologist's supervision and reintroduced only after effective management.

Study design, population, and setting/location

A prospective observational study assessed EMPA's effect on β-TM patients with T2DM against a control group on standard care. The study population consisted of patients treated at the Hereditary Blood Disorder Center of Al Karama Teaching Hospital in Baghdad, Iraq, who fulfilled the inclusion criteria. We followed a five-month study timeline from March 2024 to August 2024, with data collection between March 1 and June 4.

Inclusion and exclusion criteria

Eligible patients with β-TM and T2DM of either gender were adults (≥18 years of age) with a negative virology status for COVID-19, HIV, hepatitis C virus (HCV), and hepatitis B virus (HBV). Several exclusion criteria were applied, including the following: severely ill patients, those with a history of SGLT2 inhibitor usage or currently on insulin therapy, individuals with an estimated glomerular filtration rate (eGFR) of less than 30 mL per minute per 1.73 m² of a body-surface area or undergoing dialysis, those with fasting blood sugar (FBS) level <130 mg/dL or ≥200 mg/dL or a platelet count ≥700 × 10^9^/L. Additionally, individuals with chronic liver or renal diseases, other endocrine disorders, or those planning surgery within three months, pregnant or lactating, a medical history of cancer, drug misuse, poor medication adherence, or any condition compromising patient safety or hindering study participation were excluded.

Sampling strategy and patient enrollment

The research team employed a convenience sampling strategy because of practical constraints, including the study's observational nature in a real-world setting, accessible subjects, time sensitivity, and lack of similar studies. All screened patients were diagnosed with β-TM and T2DM, either on no oral hypoglycemic agents (OHA) or received standard care of OHA with/without lifestyle modifications, including diet and exercise. After a 10-day screening, a diabetologist evaluated eligible patients to determine suitability for EMPA prescription (Jardiance®; Boehringer Ingelheim, Ingelheim, Germany). This assessment followed current local guidelines and focused on the patient's glycemic control. This step preserved the study's observational nature and prevented bias associated with researcher-prescribed EMPA. Patients were subsequently divided into two groups: the EMPA group, which received 10 mg of EMPA orally once daily, either as first-line or add-on therapy, and the control group, which was identical in all other aspects but received alternative OHAs instead of EMPA. This design allowed for comparisons between naturally occurring groups with different drug exposures. The alternative OHAs included all FDA-approved oral antidiabetic medications, which are discussed in more detail in the Result section.

Study procedures and data collection

Both groups were monitored for three months, from March 1 to June 2, 2024. The primary objective was to assess outcomes in β-TM severity, and the secondary objective was to mitigate iron overload and hyperuricemia after 12 weeks of EMPA treatment. The impact of EMPA was evaluated based on parameters derived from two established β-TM severity scoring systems [30-32], with adjustments made by the researchers to align with the study's objectives. Consequently, the following parameters were considered: hemoglobin level, blood transfusion frequency, levels of aspartate aminotransferase (AST) and alanine aminotransferase (ALT), left ventricular ejection fraction % (EF%), and spleen size. Additionally, serum ferritin, neutrophil-to-lymphocyte ratio (NLR), and uric acid levels were measured to investigate the secondary outcome. Data for these parameters were collected at baseline, before EMPA initiation, and after three months of treatment. Patient demographics were documented through one-on-one interviews, including age, gender, splenectomy history, body mass index (BMI), T2DM duration, FBS, eGFR, type of anti-diabetic therapy, chelating therapy, and folic acid supplementation. Spleen size was assessed longitudinally using Philips IU22® ultrasound equipment (Philips Corp., Amsterdam, The Netherlands).

Statistical consideration and data analysis

All statistical analyses were conducted using SPSS (Version 26.0, IBM Corp., Armonk, NY), with significance at *P *< 0.05. Data were presented using frequencies and percentages for categorical variables, while means with standard deviations (SD) for continuous variables. The normality of distributions was assessed using the Shapiro-Wilk test and visual inspection. For pre-EMPA data, bivariate analyses employed independent t-tests for continuous variables. For post-EMPA data, a repeated-measures two-way analysis of variance (ANOVA) evaluated the effects of EMPA on β-TM severity and complications, with post hoc tests for specific group differences or time points. Effect sizes were calculated using partial eta-squared (partial η²). A sample size of 21 per group was calculated to achieve 80% power, with an effect size (δ) of 0.868 and an α error probability of 0.05. Outliers were identified with Tukey's Fences. Mauchly's test assessed sphericity, while any violations were addressed by applying Greenhouse-Geisser adjustments. The homogeneity of variances was confirmed through visual inspection of residuals.

## Results

Patient demographics and characteristics

Out of 47 patients screened for eligibility, only 20 met the criteria for study enrollment. The dropout rate primarily stemmed from factors such as noncompliance with study procedures, lack of informed consent, and geographical constraints. As a result of this sample size shortfall, the statistical power analysis indicated a decline to approximately 38.1%, elevating the risk of a type II error. Therefore, a cautious and transparent approach was taken to interpret the study findings. Patients in both groups exhibited similar baseline characteristics, with no significant differences observed in the normally distributed data, as shown in Table 1. No adverse events were recorded in either group except for uncomplicated urinary tract infections in four patients receiving EMPA. Consequently, treatment was ceased, and antibiotics were initiated. Notably, two of these incidents occurred during the third month of therapy.

**Table 1:** General profile and characteristics of patients at baseline (n = 20). BMI, body mass index; SD, standard deviation; T2DM, type 2 diabetes mellitus; FBS, fasting blood sugar; eGFR, estimated glomerular filtration rate

Variable		EMPA group (*n *= 10)	Control group (*n *= 10)
Gender	Male, *n* (%)	3 (30%)	4 (40%)
	Female, *n* (%)	7 (70%)	6 (60%)
Age (years)	Mean ± SD	36.8 ± 7.05	37.1 ± 6.39
Splenectomy	Yes, *n *(%)	2 (20%)	3 (30%)
	No, *n *(%)	8 (80%)	7 (70%)
BMI (kg/m^2^)	Mean ± SD	24.41 ± 2.86	24.44 ± 2.76
Duration of T2DM (years)	Mean ± SD	6.6 ± 4.84	5.6 ± 3.35
Baseline FBS (mg/dL)	Mean ± SD	160.7 ± 20.48	164.3 ± 17.52
Baseline eGFR (mL/min/1.73 m^2^)	Mean ± SD	60.81 ± 18.72	60.53 ± 19.05
Diabetic therapy use	Gliclazide + Metformin, *n *(%)	2 (20%)	1 (10%)
	Metformin, *n *(%)	7 (70%)	7 (70%)
	Lifestyle modifications, *n *(%)	1 (10%)	1 (10%)
	None, *n *(%)	0 (0%)	1(10%)
Chelating therapy use	Deferasirox, *n *(%)	4 (40%)	3 (30%)
	Deferoxamine, *n *(%)	4 (40%)	3 (30%)
	None, *n* (%)	2 (20%)	4 (40%)
Oral folic acid use	Yes, *n *(%)	4 (40%)	5 (50%)
	No, *n *(%)	6 (60%)	5 (50%)

Comparison of baseline characteristics between EMPA and control groups

As Table 2 shows, no significant differences were found between the EMPA and control groups in all baseline parameters.

**Table 2:** Analysis of t-test results of independent samples in the EMPA and control groups (n = 20). *EMPA group *n* = 8 and control group *n* = 7 EMPA, empagliflozin; SD, standard deviation; NLR, neutrophil-to-lymphocyte ratio; AST, aspartate aminotransferase; ALT, alanine aminotransferase; EF%, ejection fraction %

Variable	Group (Mean ± SD)		*t*- value	*P*-value
	EMPA group (*n* = 10)	Control group (*n* = 10)		
Baseline hemoglobin level (g/dL)	8.75 ± 0.64	8.72 ± 0.80	0.087	0.465
Baseline frequency of blood transfusion (times/month)	2.5 ± 0.50	2.6 ± 0.48	-0.428	0.336
Baseline serum ferritin level (ng/mL)	2334.2 ± 555.76	2481.3 ± 480.96	-0.600	0.277
Baseline serum uric acid level (mg/dL)	7.38 ± 1.04	7.05 ± 1.32	0.585	0.282
Baseline NLR	2.35 ± 0.60	2 ± 0.58	1.242	0.115
Baseline spleen size (cm)*	13.06 ± 1.87	12.97 ± 1.95	0.085	0.466
Baseline AST level (U/L)	70.61 ± 20.49	79.18 ± 19.93	-0.899	0.190
Baseline ALT level (U/L)	71.58 ± 17.14	74.03 ± 20.32	-0.276	0.392
Baseline EF%	52.6 ± 7.61	50.3 ± 6.06	0.708	0.243

In-depth analysis of EMPA impact on the β-TM outcomes

A repeated-measure two-way ANOVA was conducted to analyze the data from patients in the EMPA and control groups, and the results are summarized in Table 3. Improved hemoglobin levels over time were observed regardless of the group (FΔ = 4.728, *P *= 0.0363, partial η^2 ^= 0.120). A significant Time*Group interaction effect was noted (FΔ = 4.198, *P *= 0.047, partial η^2^ = 0.100), indicating differing magnitudes of change in hemoglobin level over time between the EMPA and control groups. However, the between-group effect was insignificant (FΔ = 0.174, *P *= 0.678, partial η2 = 0.004).

**Table 3:** Analysis of patients' response to treatment in EMPA and control groups after three months of treatment (n = 20). *Statistical significance. **EMPA group *n *= 8 and control group *n *= 7. SD, standard deviation; NLR, neutrophil-to-lymphocyte ratio; AST, aspartate aminotransferase; ALT, alanine aminotransferase; EF%, ejection fraction %

Variable	Mean ± SD		Resource	*F*-statistic	*P*-value
	After three months				
	EMPA group (*n *= 10)	Control group (*n *= 10)			
Hemoglobin level (g/dL)	9.34 ± 0.73	8.33 ± 0.61	Time	4.728	0.036*
			Group	0.174	0.678
			Time*Group interaction	4.198	0.047*
Frequency of blood transfusion (times/month)	2.2 ± 0.87	2.4 ± 0.62	Time	0.910	0.346
			Group	2.528	0.12
			Time*Group interaction	0.101	0.752
Serum ferritin level (ng/mL)	1681.8 ± 561.23	2528.7 ± 207.68	Time	9.802	0.003*
			Group	3.631	0.064
			Time*Group interaction	4.858	0.033*
Serum uric acid level (mg/dL)	5.81 ± 1.36	7.22 ± 1.72	Time	2.012	0.164
			Group	3.381	0.074
			Time*Group interaction	5.223	0.028*
NLR	1.87 ± 0.533	2.08 ± 0.526	Time	0.138	0.712
			Group	1.128	0.295
			Time*Group interaction	2.211	0.145
Spleen size (cm)**	11.42 ± 2.14	12.52 ± 2.70	Time	0.117	0.735
			Group	1.551	0.224
			Time*Group interaction	0.056	0.814
AST level (U/L)	44.99 ± 23.42	80.15 ± 20.54	Time	11.973	0.001*
			Group	3.804	0.058
			Time*Group interaction	4.426	0.042*
ALT level (U/L)	47.71 ± 17.56	74.94 ± 18.44	Time	6.009	0.019*
			Group	3.596	0.065
			Time*Group interaction	4.189	0.048*
EF (%)	53.8 ± 5.94	50.3 ± 7.82	Time	1.605	0.213
			Group	0.068	0.794
			Time*Group interaction	2.642	0.833

A group simple effects analysis was carried out, and the results (Table 4) revealed significant differences in the hemoglobin level between the EMPA and control groups (*P *= 0.877). This indicates that the hemoglobin levels in the EMPA group were significantly greater than those of the control group after three months of treatment.

**Table 4:** Simple effects analysis of group between the EMPA group and control group before and after the intervention (n = 20). *Statistical significance AST, aspartate aminotransferase; ALT, alanine aminotransferase; EMPA, empagliflozin

Variable	Time	Group	Average difference	Standard error	*P*-value
Hemoglobin level	Baseline	EMPA group - Control group	0.03	-0.16	0.877
	Three months	EMPA group - Control group	1.01	0.12	0.005*
Serum ferritin level	Baseline	EMPA group - Control group	-147.1	74.8	0.836
	Three months	EMPA group - Control group	-846.9	353.55	0.028*
Serum uric acid	Baseline	EMPA group - Control group	0.33	-0.28	0.836
	Three months	EMPA group - Control group	-1.41	-0.36	0.024*
AST level	Baseline	EMPA group - Control group	-8.57	0.56	0.784
	Three months	EMPA group - Control group	-26.62	8.202	0.025*
ALT level	Baseline	EMPA group - Control group	1.95	7.6	0.623
	Three months	EMPA group - Control group	-27.23	-0.88	0.017*

The serum ferritin significantly decreased over time, regardless of the group (FΔ = 9.802, *P *= 0.003, partial η^2^ = 0.21). Although the between-group effect was insignificant (FΔ = 3.631, *P *= 0.064, partial η^2^ = 0.092], the Time*Group interaction effect was significant (FΔ = 4.858, *P *= 0.033, partial η^2 ^= 0.120), indicating differences in the serum ferritin over time between the EMPA and control groups. Subsequently, a group simple effects analysis was conducted, revealing significant differences between the EMPA and control groups (*P *= 0.028), as shown in Table 4.

**Figure 1:**
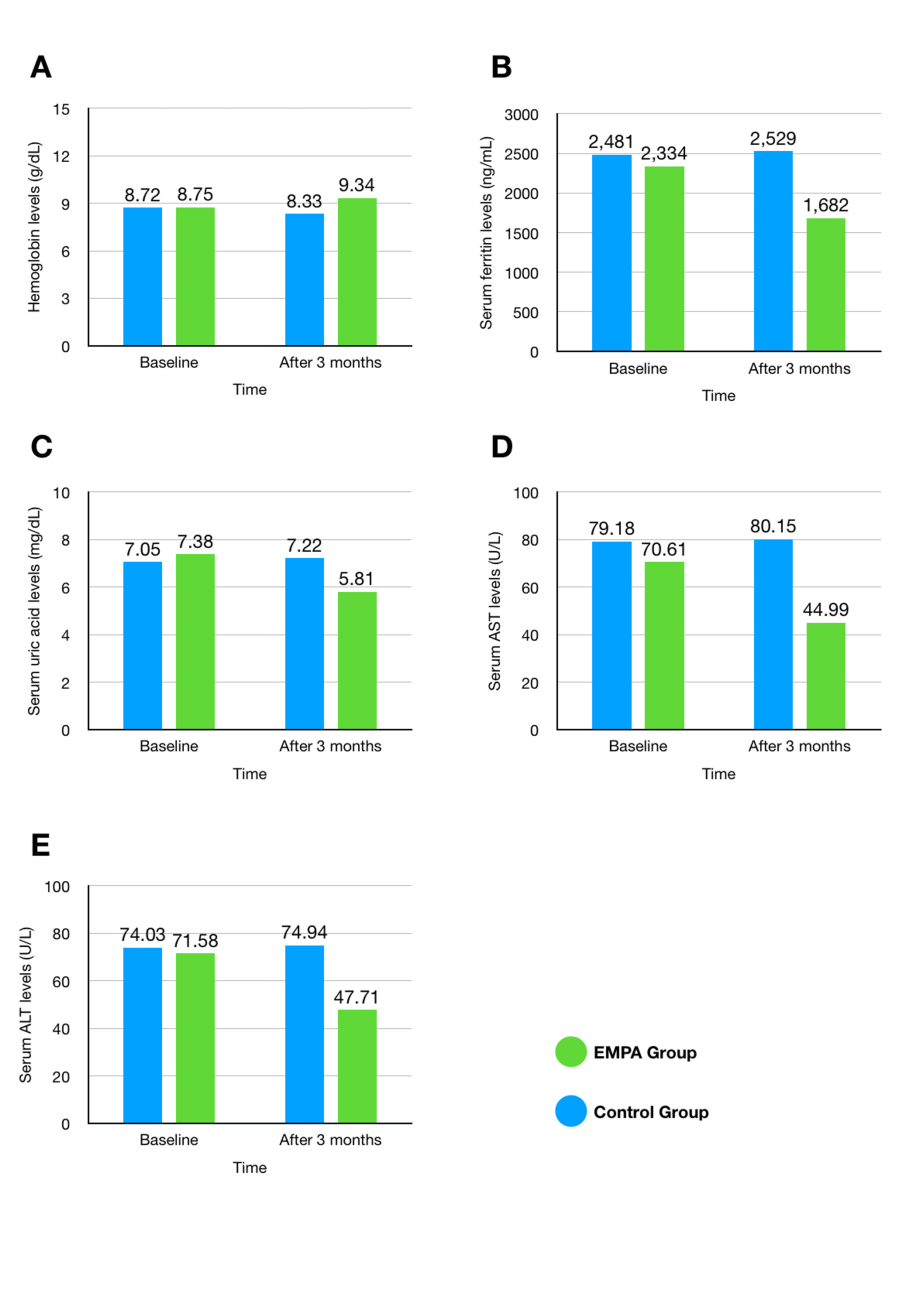
Visualization of the means of statistically significant parameter changes between the EMPA and control groups (n = 20) over three months. (A) Hemoglobin levels; (B) serum ferritin levels; (C) serum uric acid levels; (D) serum aspartate aminotransferase (AST) levels; (E) serum alanine aminotransferase (ALT) levels.

Although the time and between-group effects were not statistically significant for uric acid (FΔ = 2.012, *P *= 0.164, partial η^2^ = 0.053) and (FΔ = 3.381, *P* = 0.074, partial η^2 ^= 0.086), respectively, the Time*Group interaction effect was significant (FΔ = 5.223, *P *= 0.028, partial η^2^ = 0.130). This indicates that the EMPA and control groups exhibited different patterns of change in uric acid levels over time. Subsequently, a group simple effects analysis was conducted, revealing significant differences in uric acid levels between the EMPA and control groups after three months (*P *= 0.024), as shown in Table 4. The liver function tests showed significant improvement over time, irrespective of the group: AST level (FΔ = 11.973, *P *= 0.001, partial η^2^ = 0.250) and ALT level (FΔ = 6.009, *P* = 0.019, partial η2 = 0.14). While the between-group effect was insignificant for both ALT and AST levels, the Time*Group interaction effect was significant: AST level (FΔ = 4.426, *P *= 0.042, partial η^2^ = 0.110) and ALT level (FΔ = 4.189, *P* = 0.048, partial η^2^ = 0.100). This indicates that the EMPA and control groups differed in the extent of change in liver function over time. Subsequently, a simple group effects analysis was conducted, revealing significant differences in liver function between the EMPA and control groups in months two and three for both AST and ALT, as shown in Table 4. The graphs in Figure 1 provide a clearer visualization of the three-month changes in the statistically significant parameters.

## Discussion

The key findings of the present study demonstrate that the selective SGLT2 inhibitor, EMPA, improves hemoglobin levels, liver enzymes, serum ferritin, and hyperuricemia in a small-scale clinical observational study involving β-TM patients diagnosed with T2DM. We illustrated that EMPA led to a rise in hemoglobin levels, reaching up to 1.01 g/dL average difference by the end of the third month compared to the control group and 0.56 g/dL from the baseline EMPA group. These findings are consistent with several studies that investigated the impact of SGLT2 inhibitors on hematological markers [33-35]. In particular, Mazer et al. reported that a daily 10 mg EMPA increased hemoglobin levels by 0.583 g/dL and hematocrit levels by 2.34% after six months of treatment compared to baseline [18]. Although the latter study used the exact dosage as ours, the study duration was extended to six months, and participants did not have thalassemia. It is crucial to note that the EMPA effect is dose and duration-dependent [20].

The mechanism underlying the induction of hemoglobin and hematocrit levels by SGLT2 inhibitors is currently understood to be multifaceted. This includes increased erythropoietin (EPO) production by increasing the afferent arteriolar vasoconstriction, enhancing the expression of hypoxia-inducible factor, and suppressing hepcidin with modulation of iron metabolism. Finally, the diuretic effect of SGLT2 inhibitors causes hemoconcentration, resulting in higher concentrations of red blood cells and hemoglobin [20]. While these rationales provide valuable insights, they may not fully elucidate the mechanism behind the elevated hemoglobin levels observed in our study, particularly considering that ineffective erythropoiesis is a characteristic feature of β-thalassemia patients [1]. Given the lack of prior research exploring the effects of SGLT2 inhibitors on thalassemia patients, it's crucial to acknowledge the potential for exacerbating preexisting extramedullary hematopoiesis. This phenomenon could further complicate complications such as organomegaly, bone deformities, and fascial changes in these patients. This topic fell outside the scope of our study; therefore, additional research must be undertaken to investigate this matter thoroughly.

Based on the study's findings, no significant difference was observed in the rate of blood transfusions in the EMPA group. Although higher hemoglobin levels correlate inversely with a lower rate of transfusions, it's essential to acknowledge that various factors determine the transfusion rate [36-38]. These factors, such as facial changes, growth retardation, spontaneous fractures, and clinically significant extramedullary hematopoiesis, were not taken into consideration in this study, which may explain the steady rate of transfusion in our patients. Additionally, the short duration of the research and the small sample size might also contribute to these limitations.

Iron overload declined with EMPA, resulting in approximately a 27.938% reduction in serum ferritin levels from baseline. Similarly, a study by Reppo et al. reported a significant reduction in serum ferritin of 11.9% after three months of EMPA compared to semaglutide in patients with T2DM [34]. Mazer et al. reported a reduction of 17.4% in serum ferritin compared to baseline in patients with T2DM with cardiovascular risk [18]. Despite the anti-inflammatory properties of SGLT2 inhibitors that may explain the decrease in serum ferritin [39], our research suggests that the reduction in ferritin levels indicates an improvement in iron overload. We arrived at this assumption because we did not observe any significant difference in the levels of NLR, an inflammatory marker, after three months of EMPA. However, the small sample size and low statistical power may contribute to a heightened risk of failing to detect the genuine anti-inflammatory effects of SGLT2 inhibitors. Therefore, additional research is necessary to validate this assumption in thalassemia patients known for their high ferritin levels and risk of iron overload.

The present study observed a statistically significant decline in uric acid levels in the EMPA group to approximately 21.29% compared to baseline. These findings align with prior research indicating a 10%-15% reduction in uric acid following EMPA treatment [34,40]. SGLT2 inhibitors have been proposed to lower uric acid levels by enhancing urinary excretion, achieved through reducing reactive oxygen species and inhibiting xanthine oxidase enzyme activity [41]. Furthermore, hyperglycosuria induced by SGLT2 inhibitors may promote the secretion of uric acid in exchange for glucose reabsorption through the glucose transporter 9 [28]. Despite recent recognition of uric acid as an inflammatory factor [27], the insignificant change in NLR suggests that the reduction in uric acid levels is more closely linked to increased urinary excretion than a decreased inflammatory status. This assumption requires further research for validation, as previously discussed.

The hyperuricosuria induced by SGLT2 inhibitors presents an additional risk for renal stones, a condition that is not uncommon in thalassemia patients [42]. While this aspect was not directly examined in our study, it warrants careful consideration. Specifically, hyperuricosuria, in conjunction with the hyperglycosuria effect of SGLT2 inhibitors, synergistically contributes to the development of urinary tract infections [43], as was evident by occurrences of urinary tract infections in four patients in the EMPA group.

Liver enzyme levels demonstrated improvement by the third month in the EMPA group, with AST and ALT experiencing a maximum decline of 36.22% and 33.36%, respectively, from the average baseline levels. These findings align with the Kuchay et al. study on patients with T2DM and non-alcoholic fatty liver disease (NAFLD) [44]. Whereas the mechanism behind this effect on liver function is still debated, Zhang Y et al. suggested it could result from a direct effect of EMPA on reducing liver fat, insulin resistance, and liver fibrosis and enhancing hepatic glucose handling [45]. This assumption in our study is limited, as we did not monitor glycemic control or liver fibrosis. Another potential explanation for this improvement in transaminase levels is the anti-inflammatory properties of EMPA [39], which is under debate in our study.

We propose that a potential mechanism underlying the improvement of transaminase levels involves reducing iron overload within hepatocytes, as evidenced by the decrease in serum ferritin levels. Nevertheless, further research is necessary to validate this hypothesis across diverse populations. This could involve relying on a reliable quantitative technique for iron overload, such as MRI T2*, instead of solely relying on serum ferritin [46]. However, if advanced imaging modalities are unavailable, including more inflammatory markers like high-sensitivity CRP, procalcitonin, and IL6 could be suitable alternatives. Adjusting for these relevant covariates can enhance the accuracy and generalizability of study findings.

In this study, EMPA did not significantly affect spleen size. No human studies have directly investigated how SGLT2 inhibitors affect the spleen. However, evidence from rat research suggests a notable reduction in spleen weight associated with EMPA, possibly due to reductions in visceral obesity or alterations in the immune system [47,48]. This underscores the need for further research in this area. Although SGLT2 inhibitors have proven beneficial for cardiovascular outcomes in various studies [14-17], we did not observe a similar improvement in the EF% in our research. This unexpected outcome may be attributed to the small sample size, short study duration, and confounding variables, including the absence of heart failure diagnosis in all study participants. On the other hand, dismissing the potential cardiovascular benefits of EMPA based solely on one parameter (i.e., EF%) would be unfair. It's important to note that our study's objective did not encompass evaluating other parameters such as heart rate, blood pressure, exercise intolerance, and biomarkers like brain natriuretic peptide (BNP) or N-terminal pro-b-type natriuretic peptide (NT-proBNP).

Several recommendations can be considered for future research to overcome the limitations encountered in this study, although it was a prospective design with a comparative control group. A larger sample size, longer duration, and adequate statistical power can help detect more minor treatment effects and reduce the likelihood of type II errors. Moreover, a randomized controlled trial with appropriate blinding can minimize bias and enhance internal validity.

## Conclusions

The current study investigates the impact of three-month treatment with EMPA on the β-TM outcomes in patients with T2DM. Our findings reveal that EMPA effectively improves the severity of β-TM, as evident in hemoglobin, AST, and ALT levels. However, we observed a neutral effect on blood transfusion frequency, spleen size, and EF%. Notably, EMPA significantly reduces β-TM complications of iron overload and hyperuricemia. Future research should focus on confirming these benefits over longer durations, exploring EMPA's mechanisms of action, integrating it into clinical guidelines for β-TM with T2DM, and assessing broader patient outcomes such as quality of life and glycemic control through larger-scale trials.

## Figures and Tables

**Figure 2:**
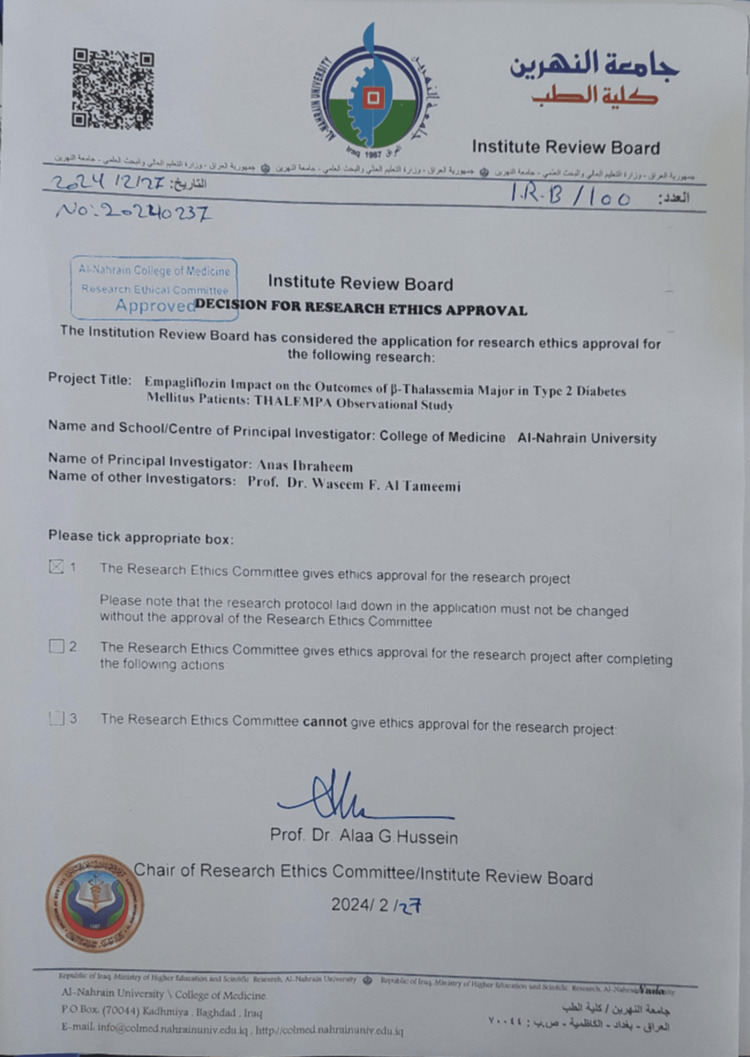
Institution Review Board Approval Letter
